# Ignore the faces: Neural characterisation of emotional inhibition from childhood to adulthood using MEG

**DOI:** 10.1002/hbm.25651

**Published:** 2021-09-28

**Authors:** Marlee M. Vandewouw, Kristina Safar, Julie Sato, Benjamin A. E. Hunt, Charline M. Urbain, Elizabeth W. Pang, Evdokia Anagnostou, Margot J. Taylor

**Affiliations:** ^1^ Department of Diagnostic Imaging Hospital for Sick Children Toronto Canada; ^2^ Program in Neurosciences & Mental Health Hospital for Sick Children Toronto Canada; ^3^ Autism Research Centre, Bloorview Research Institute Holland Bloorview Kids Rehabilitation Hospital Toronto Ontario Canada; ^4^ Institute of Biomedical Engineering University of Toronto Toronto Canada; ^5^ Department of Psychology University of Toronto Toronto Canada; ^6^ Neuropsychology and Functional Neuroimaging Research Group at CRCN, Center for Research in Cognition and Neurosciences ULB Neurosciences Institute, Université Libre de Bruxelles Brussels Belgium; ^7^ Laboratoire de Cartographie Fonctionnelle du Cerveau, ULB Neuroscience Institute Université Libre de Bruxelles Brussels Belgium; ^8^ Division of Neurology Hospital for Sick Children Toronto Canada; ^9^ Department of Medical Imaging University of Toronto Toronto Canada

**Keywords:** automatic emotion regulation, development, emotional go/no‐go, magnetoencephalography

## Abstract

The ability to effectively and automatically regulate one's response to emotional information is a basic, fundamental skill for social functioning. The neural mechanisms underlying emotion regulation processing have been assessed, however few investigations have leveraged neurophysiological techniques, particularly magnetoencephalography (MEG) to determine the development of this critical ability. The current MEG study is the first to examine developmental changes in the neural mechanisms supporting automatic emotion regulation. We used an emotional go/no‐go task with happy and angry faces in a single‐site cohort of 97 healthy participants, 4–40 years of age. We found age‐related changes as a function of emotion and condition in brain regions key to emotion regulation, including the right inferior frontal gyrus, orbitofrontal cortices and primarily right‐lateralized temporal areas. Interaction effects, including an age by emotion and condition, were also found in the left angular gyrus, an area critical in emotion regulation and attention. Findings demonstrate protracted and nonlinear development, due to the adolescent group, of emotion regulation processing from child to adulthood, and highlight that age‐related differences in emotion regulation are modulated by emotional face type.

## 1 INTRODUCTION

The ability to regulate our emotions and respond to experiences with the appropriate emotion, intensity and duration is fundamental to successful social interactions (Gross, 2013). Humans have a range of automatic emotion regulation strategies to cope with the otherwise resource‐demanding conscious task of processing emotionally charged stimuli (Braunstein, Gross, & Ochsner, 2017; Gyurak, Gross, & Etkin, 2011; Koole & Rothermund, 2011). Although a considerable literature has focussed on explicit emotion regulation, automatic regulation is increasingly recognised as an important life skill to prevent distraction from an almost constant stream of emotional stimuli. During automatic emotion regulation (Mauss, Bunge, & Gross, 2007), emotional cues can be rapidly detected while simultaneously inhibiting potential responses to limit the impact of the emotional experience on one's ongoing activity (Koole, Webb, & Sheeran, 2015). For successful interpersonal social functioning, being able to effectively process emotional stimuli while simultaneously inhibiting one's response is a vital, acquired skill (De France & Hollenstein, 2019).

Researchers have reported remarkable developmental changes in emotion regulation abilities through childhood and adulthood (for reviews see: Benson et al., 2019; Todd & Lewis, 2008). Generally studies show increasing abilities in emotion regulation with increasing age, and that negative emotions impact emotion regulation more than positive emotions (Ahmed, Bittencourt‐Hewitt, & Sebastian, 2015; Zhang et al., 2016). Several studies also show, however, nonlinear trajectories, with poorer performance in adolescence, suggesting it is a particularly vulnerable period for this skill acquisition (e.g., Ahmed et al., 2015; Cohen‐Gilbert & Thomas, 2013; Lewis, Lamm, Segalowitz, Stieben, & Zelazo, 2006), which has led to studies focussed specifically on the adolescent years (Schweizer, Gotlib, & Blakemore, 2020; Zhang et al., 2016). Despite the importance of developing emotion regulation abilities over childhood and adolescence, there are few studies that have examined the neural underpinnings across these age groups (e.g., Lewis et al., 2006; Somerville, Hare, & Casey, 2011), and only a few that have looked at automatic emotion regulation.

Neuroimaging has been invaluable in determining the neural mechanisms of both emotional face processing and response inhibition. Studies have established that face processing relies on a core system consisting of the inferior occipital gyri, superior temporal sulci and fusiform gyri, with involvement from the amygdalae, insulae and limbic systems to process emotions (for a detailed review see Haxby, Hoffman, & Gobbini, 2000). Go/no‐go tasks have been used to study response inhibition, in which participants are presented with stimuli requiring rapid responses interleaved with stimuli to which responses are to be inhibited. These studies have identified the orbitofrontal and prefrontal cortices (e.g., Hege, Preissl, & Stingl, 2014) and inferior frontal gyri (IFG; Aron, Robbins, & Poldrack, 2014; Chikazoe, 2010; Swick, Ashley, & Turken, 2008) as critical to this cognitive control process. The intersection of these two social cognitive processes, emotional face processing and response inhibition, has been examined to probe the nature of the important, adaptive skill of automatic emotion regulation, usually using go/no‐go tasks with emotional faces. Emotional go/no‐go tasks recruit the regions typically involved in response inhibition in adults, particularly the right IFG, while also relying on common emotional face processing areas, the inferior parietal and temporal regions, the anterior cingulate and the insula (Albert, López‐Martín, & Carretié, 2010; Brown et al., 2012; Goldstein et al., 2007; Schulz et al., 2009; Shafritz, Collins, & Blumberg, 2006; Taylor et al., 2018).

Previous neuroimaging studies using an emotional go/no‐go task have employed predominantly functional magnetic resonance imaging (fMRI). Somerville et al. (2011) reported fMRI data on happy versus neutral faces (the fearful faces were published separately (Hare et al., 2008)) in children, teens and adults, where decreases were seen with age in the right IFG, but other regions showed nonlinear effects, with the teens showing the opposite pattern to children and adults. An fMRI go/no‐go task including young children and adults and happy and angry faces, Todd, Lee, Evans, Lewis, and Taylor (2012) found that inhibition‐related orbito‐frontal activity was modulated by emotion in children across the 4–9‐year age range.

However, the poor temporal resolution of fMRI prevents investigation of the timing of related brain dynamics, while electroencephalography (EEG) has good temporal resolution but coarse spatial resolution (Somerville et al., 2011; Zhang et al., 2016). Magnetoencephalography (MEG) is a neuroimaging modality which provides similar, excellent temporal resolution as EEG but higher spatial resolution (Hari & Salmelin, 2012). The temporally enriched MEG data facilitate the examination of transient neural oscillations in different frequency bands, known to reflect different cognitive processes (Palva, Monto, & Palva, 2010; Pfurtscheller & Lopes Da Silva, 1999; Zhigalov, Arnulfo, Nobili, Palva, & Matias Palva, 2015). Thus, while fMRI provides low resolution temporal measures of neural activity using the blood‐oxygen‐level‐dependent (BOLD) signal, the temporally rich MEG data gives greater insight into the neural processes that arise during cognitive tasks such as emotion regulation (Lopes da Silva, 2013; Singh, 2012) and hence is better able to characterise its development throughout the lifespan.

Using a go/no‐go task with faces in 7–13‐year‐olds and MEG, Urbain, Sato, Pang, and Taylor (2017) found greater activity in the first 400 ms following the angry emotional faces in children; the areas involved included the orbital frontal gyrus, temporal pole, as well as angular and occipital gyri. With the same emotional faces go/no‐go MEG paradigm in adults (Taylor et al., 2018), the right IFG showed sustained activity (200–450 ms post stimulus onset) during inhibition regardless of emotion. Inhibition during emotion processing showed increased activation to angry faces in the orbitofrontal gyri and temporal regions, with happy faces showing increased activity earlier in the right orbitofrontal gyrus (200 ms).

Behavioural and neuroimaging studies have demonstrated that automatic emotion regulation continues to develop into early adulthood (see Ahmed et al., 2015, for a review), coinciding with the protracted development in key brain regions: the limbic system and prefrontal cortex (Cloak et al., 2010; Gee et al., 2013; Gogtay et al., 2004; Schumann et al., 2004). To date, no study has examined automatic emotional regulation in a large sample spanning children, adolescents and adults to investigate how the neurophysiological brain dynamics change with age. Establishing typical trajectories of automatic emotion regulation is a key first step to identifying potential deviations in clinical populations who present with emotion regulation difficulties (e.g., Christiansen, Hirsch, Albrecht, & Chavanon, 2019; Eack et al., 2016; Mazefsky et al., 2013; Park et al., 2019).

In the current study, we used MEG to investigate how the neural mechanisms supporting automatic emotion regulation change with age in a cohort of 97 typical participants 4–40 years of age. We used an emotional go/no‐go task with happy and angry faces to investigate whether emotional face type modulated inhibitory activity. We hypothesised increases in frontal activity during inhibition with age, particularly the IFG, that would extend beyond adolescence, consistent with the model for protracted development of inhibitory control (Vara, Pang, Vidal, Anagnostou, & Taylor, 2014) and that age‐related changes would be greater in the presence of angry faces.

## 2 METHODS

### 2.1 Participants

One hundred and fifty‐three participants ranging from 4 to 40 years of age (56 children [4–10 years], 48 adolescents [11–19 years] and 49 adults [20–40 years]) were recruited at the Hospital for Sick Children (Toronto, Canada), approved by the hospital's Research Ethics Board and in accordance with relevant guidelines and regulations. All participants underwent the emotional go/no‐go MEG scanning protocol, some of whom (43% of the sample) were included in our previous emotional go/no‐go studies (Taylor et al., 2018; Urbain et al., 2017). Participants who were born premature or who had been diagnosed with learning, language, neurological or developmental disabilities were excluded from these analyses. Informed consent was provided by participants who were old enough to do so; otherwise, informed assent was obtained from the participant and informed written consent from the caregiver/parent.

### 2.2 Emotional go/no‐go task: further details

The emotional go/no‐go task consisted of two conditions: vigilance and inhibition, performed as separate runs. During each condition, participants were presented with a randomised series of happy and angry faces from a subset of 52 individuals (26 female, 26 male) selected from the NimStim Set of Facial Expressions (Tottenham et al., 2009) whose emotion classification accuracy exceeded 80%. The stimuli were formatted to 7.4 × 9 cm rectangle and surrounded by a 1 cm blue or purple border, and presented at a visual angle of 5.5 × 9° approximately 80 cm from the participants' eyes at a luminance of 65 Lux. Participants were instructed to respond via button press as quickly as possible when presented with a target colour (considered ‘go’ stimuli) and refrain from responding when presented with the alternate colour (considered ‘no‐go’ stimuli), while ignoring the face within the frame. This allowed the investigation of the impact of emotional valence on response inhibition. The vigilance condition consisted of 25% go trials while the inhibition condition consisted of 75% go trials, creating a prepotent tendency to respond and thus producing an increased inhibitory load. Stimulus duration was adjusted between 300 and 500 ms to maintain steady error rates (≥95% for go trials, ≥80% for no‐go trials), and followed by an interstimulus interval consisting of a fixation cross, which was also adjusted between 650 and 1,300 ms according to error rates. The assigned target colour and the order in which the two conditions were presented were randomised across participants. Reaction times were extracted from the go trials. For all analyses, however, only the no‐go trials were analysed to avoid the motor confound associated with the go‐trials, and participants who did not perform above chance (>55% accuracy in the no‐go trials) were excluded from the analyses.

### 2.3 Image acquisition

Continuous MEG data were recorded at a 600 Hz sampling rate using a 151‐channel CTF system (CTF MEG Neuro Innovations Inc., Coquitlam, B.C., Canada) within a magnetically shielded suite. To improve signal quality, a third‐order spatial gradient was used with a recording bandpass of 0–150 Hz for anti‐aliasing. Head location was continuously monitored using fiducial coils placed at the bilateral pre‐auricular points and the nasion. After recordings, radio‐opaque markers replaced the fiducial coils and participants also completed MRI scanning to allow for MEG‐MRI co‐registration. A T1‐weighted image was obtained on one of two 3.0T MAGNETOM Siemens scanners (due to a scanner upgrade): a Trio with a 12‐channel head coil (TR/TE = 2300/2.96 ms, FA = 9°, FOV = 240 × 256 mm, # slices = 192, resolution = 1.0 mm isotropic), or a PrismaFIT with a 20‐channel head and neck coil (TR/TE = 1870/3.14 ms, FA = 9°, FOV = 240 × 256 mm, # slices = 192, resolution = 0.8 mm isotropic).

### 2.4 MEG preprocessing

MEG data were processed using the FieldTrip software toolbox (Oostenveld, Fries, Maris, & Schoffelen, 2011). Data were filtered between 1 and 150 Hz using a fourth order two‐pass Butterworth filter, and line noise was eliminated using a discrete Fourier transform notch filter at 60 and 120 Hz. Data were epoched into trials from −1,000 ms to 1,500 ms relative to the stimulus onset and mean‐centred; only correct no‐go trials were retained. Trials where head movement exceeded 10 mm from the trial's median head position were excluded; this threshold was chosen to conform with MEG studies involving children (Doesburg, Vidal, & Taylor, 2013; Pang, 2011; Safar, Wong, Leung, Dunkley, & Taylor, 2018). Independent component analysis was employed to remove trials contaminated by artefacts such as heartbeats, eye blinks and eye movements, and trials with MEG sensor signals exceeding 2000fT. Only participants with at least 20 trials remaining for each emotion and condition were retained. The vigilance condition was significantly easier for the participants than the inhibition condition, and thus more correct trials remained. To prevent confounds due to unequal numbers of trials per condition, the number of trials was matched across condition (within‐emotion) for each participant by dropping excess vigilance trials.

### 2.5 Source reconstruction

The MEG data were co‐registered to each participant's MRI, and the MRI was used to construct a subject‐specific single‐shell head model. The coordinates of an 8 mm grid in MNI space were unwarped into each participant's head space, and a linear constrained minimum variance (LCMV) beamformer (Van Veen, Van Drongelen, Yuchtman, & Suzuki, 1997) with 5% Tikhonov regularisation was used to compute a single common spatial filter for each participant based on the covariance of all selected trials in each condition. The data were projected through the spatial filter to estimate the timeseries for source locations across a 8 mm grid for each trial, corrected for centre‐of‐head bias using the Neural Activity Index and broadband filtered between 1 and 50 Hz. The Hilbert transform was applied to the filtered timeseries to extract instantaneous power between −100 and 500 ms relative to stimulus onset for the source locations for each trial, converted to percentage change from baseline (−100 to 0 ms), and averaged over emotion for each condition. Instantaneous power was reduced to the 90 ROIs in the automated anatomical labelling (AAL) atlas (Tzourio‐Mazoyer et al., 2002) by averaging across all 8 mm voxel sources belonging to each ROI, weighted by the distance to the centroid using a Gaussian weighting function, to ensure the resulting timeseries were biased towards its centroid (Brookes et al., 2016). Timeseries were then averaged over sliding time windows (50 ms long, 25 ms overlap, between 100 and 500 ms) to pinpoint the onset and offset of brain activity. Details on the AAL regions can be found in Table S1. The LCMV beamformer has been shown to accurately estimate source power in comparison to other techniques (Hincapié et al., 2017), and achieves a spatial resolution of <5 mm in conditions with a reasonable signal‐to‐noise ratio (Lin, Witzel, Zeffiro, & Belliveau, 2008).

### 2.6 Statistics: participant demographics

A chi‐squared test was used to ensure no significant difference in the proportion of males and females across the three age groups (children, adolescents and adults). Repeated measure analysis of variances (ANOVAs) were employed to ensure no significant differences among the three age groups in mean head motion and the number of trials included in the final analyses. Both condition (inhibition/vigilance) and emotion (happy/angry) were used as the within‐subject factors for mean head motion, and age group as the between‐subject factor; due to the number of trials being matched across conditions only emotion (happy/angry) was used as the within‐subject factor for this measure.

Repeated‐measure ANOVAs were also used to evaluate the performance measures (reaction time for the go trials and accuracy for the go and no‐go trials) with condition (inhibition/vigilance) and emotion (happy/angry) as within‐subject factors; significance was held at *p* < .05. Upon significance of an interaction, post‐hoc Fisher's least significant difference procedure was used to determine the directionality of effects, with significance held at *p* < .05. For effect sizes, partial eta‐square values were reported for the omnibus tests, while differences in means (MD) with 95% confidence intervals were reported for the post‐hoc tests. This procedure was also followed for the stimulus durations, and results are presented in Table S2 and the subsequent text.

For each time window, the power data for each of the 90 regions were entered into repeated measures ANOVAs to examine age group‐by‐emotion, age group‐by‐condition, and age group‐by‐condition‐by‐emotion interactions; sex was included as a covariate. For the age group‐by‐emotion interaction, only the vigilance condition was used to avoid the potential confound of inhibition. For each interaction, *p*‐values were FDR‐corrected (Benjamini & Hochberg, 1995) across the 90 AAL regions and number of pairwise comparisons, and significance was held at *p*_corr_ < .05. Upon significance of an AAL region, the significant time windows were aggregated to establish precise onset and offset of significance and the *F*‐statistic and *p*_corr_‐values were reported for the time window with the maximum *F*‐statistic. Post‐hoc Fisher's least significant difference procedures were used to identify the directionality of effects.

## 3 RESULTS

### 3.1 Participants

After removing participants who failed to meet the strict task inclusion criteria, data from 97 of the original 153 participants remained: 25 children (4–10 years), 32 adolescents (11–19 years), and 40 adults (20–40 years). There was no significant difference in the proportion of males and females among the age groups (*χ* ^2^ = 1.13, *p* = .56). There were no main effects of age group (*F*(2,113) = 2.31, *p* = .10), condition (*F*(2,95) = 1.08, *p* = .32) or emotion (*F*(2,95) = 0.28, *p* = .60) on mean head motion, nor age group‐by‐condition (*F*(2,94) = 1.19, *p* = .31), age group‐by‐emotion (*F*(2,94) = 0.86, *p* = .43), condition‐by‐emotion (*F*(2,95) = 0.17, *p* = .68), or age group‐by‐condition‐by‐emotion (*F*(2,94) = 1.31, *p* = .27) interactions for motion. Furthermore, there was no effect of age group (*F*(2,94) = 1.92, *p* = .15), emotion (*F*(2,95) = 0.04, *p* = .84), nor an age group‐by‐emotion interaction (*F*(2,94) = 2.34, *p* = .10) on the number of trials analysed (since the number of trials were matched across condition, it was not used as a within‐subject factor). Descriptive statistics of the participants are summarised in Table 1.

**TABLE 1:** Participant demographics (sample size, age, proportion of males and females), along with descriptive statistics of the mean head motion and number of trials for both conditions (INH: inhibition, VIG: vigilance) and emotions (H: happy, A: angry) in the three age groups

	Children	Adolescents	Adults
	(4–10 years)	(11–19 years)	(20–40 years)
*N*	25	32	40
Mean age (years ± std.)	8.38 ± 1.48	14.23 ± 2.61	28.13 ± 5.14
Sex (M:F)	14:11	20:12	20:20
Mean head motion (mm ± std.)			
VIG, H	0.61 ± 0.18	0.49 ± 0.29	0.44 ± 0.43
VIG, A	0.56 ± 0.15	0.53 ± 0.38	0.44 ± 0.42
INH, H	0.66 ± 0.2	0.51 ± 0.28	0.46 ± 0.43
INH, A	0.65 ± 0.21	0.49 ± 0.27	0.45 ± 0.43
Mean # of trials (± std.)			
H	26.84 ± 5.98	28.69 ± 5.02	27.83 ± 2.33
A	26.24 ± 5.45	28.22 ± 4.12	28.70 ± 3.09

*Note*: *N*: number of participants, std: standard deviation, M: male, F: female, VIG: vigilance, INH: inhibition, H: happy, A: angry.

### 3.2 Performance measures

**FIGURE 1:**
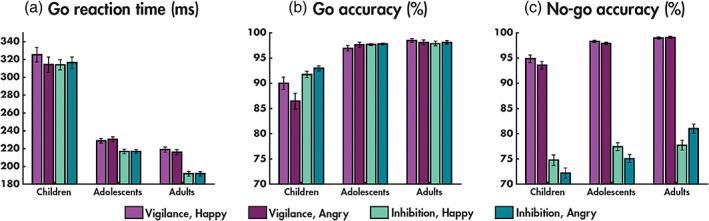
Performance measures. Reaction times for the go trials (a), accuracy for the go trials (b), and accuracy for the no‐go trials (c) for each condition (vigilance, inhibition) and emotion (happy, angry) pair, for the three age groups (children, adolescents and adults)

There was a significant main effect of age group in reaction time (RT) to the go trials (*F*(2,94) = 74.34, *p* = 4.36 × 10^−20^; *η*_*p*_^2^ = 0.613) and to condition (*F*(1,153) = 14.47, *p* = 2.53 × 10^−4^; *η*_*p*_^2^ = 0.133) (Figure 1a; Table 2). Post‐hoc tests showed that across age groups, RTs were faster during the inhibition compared to vigilance condition (*p* = 2.53 × 10^−4^; MD = 14.41, CI_95%_ = [6.89, 21.92]), and children had significantly slower RTs across conditions and emotions compared to the adolescents (*p* = 3.14 × 10^−15^; MD = 94.33, CI_95%_ = [74.44, 114.21]) and adults (*p* = 2.96 × 10^−20^; MD = 112.86, CI_95%_ = [93.88, 131.85]), and adolescents responded more slowly than the adults (*p* = .04; MD = 18.54, CI_95%_ = [0.87, 36.21]). There was no significant main effect of emotion (*F*(2,95) = 1.42, *p* = .24; *η*_*p*_^2^ = 0.015), nor age group‐by‐emotion (*F*(2,94) = 0.99, *p* = .38; *η*_*p*_^2^ = 0.021), age group‐by‐condition (*F*(2,94) = 2.70, *p* = .07; *η*_*p*_^2^ = 0.054), condition‐by‐emotion (*F*(2,95) = 2.86, *p* = .09; *η*_*p*_^2^ = 0.029), or age group‐by‐condition‐by‐emotion interactions (*F*(2,94) = 2.04, *p* = .14; *η*_*p*_^2^ = 0.042) on RTs.

**TABLE 2:** Statistics related to the performance measures showing significant (*p*_corr_ < .05) main effects and interactions. Results of the post‐hoc tests are provided to show the directionality of effects

	*F*‐statistic	*p*_corr_	*η*_p_^2^	Post‐hocs (for *p*‐values see Table S3)											
**A. Main effect of age group**				**C > Ad**		**Ad > C**		**C > AD**		**AD > C**		**AD > AD**		**AD > Ad**	
Reaction time	73.34	4.36 × 10^−20^	0.613	X				X				X			
Go accuracy	17.13	4.54 × 10^−7^	0.267			X				X					
No‐go accuracy	8.57	3.8 × 10^−4^	0.154			X				X					
**B. Main effect of condition**				**V > I**						**I > V**					
Reaction time	14.47	2.53 × 10^−4^	0.133	X											
Go accuracy	7.08	0.01	0.070							X					
No‐go accuracy	731.78	3.84 × 10^−46^	0.886	X											

				Children				Adolescents				Adults			
Age group × condition				V > I		I > V		V > I		I > V		V > I		I > V	
Go accuracy	4.67	0.01	0.090			X									

				Children				Adolescents				Adults			
Age group × emotion				H > A		A > H		H > A		A > H		H > A		A > H	
No‐go accuracy	6.42	2.4 × 10^−3^	0.120	X								X			

				H						A					
Condition × emotion				V > I			I > V			V > I			I > V		
Go accuracy	4.90	0.03	0.050										X		

				Children				Adolescents				Adults			
				H > A		A > H		H > A		A > H		H > A		A > H	
Age group × condition × emotion				V	I	V	I	V	I	V	I	V	I	V	I
No‐go accuracy	4.06	0.02	0.079	X											X

*Note*: C: children, Ad: adolescents, AD: adults, V: vigilance, I: inhibition, H: happy, A: angry.

There were main effects of age group (*F*(2,94) = 17.13, *p* = 4.54 × 10^−7^; *η*_*p*_^2^ = 0.267) and condition (*F*(2,95) = 7.08, *p* = 9.17 × 10^−3^; *η*_*p*_^2^ = 0.070), and age group‐by‐condition (*F*(2,94) = 4.67, *p* = .01; *η*_*p*_^2^ = 0.090) and condition‐by‐emotion (*F*(2,95) = 4.90, *p* = .03; *η*_*p*_^2^ = 0.050) interactions on the accuracy of the *go trials* (Figure 1b; Table 2). Post‐hoc tests found that the children had significantly worse accuracy across conditions and emotions compared to the adolescents (*p* = 1.71 × 10^−6^; MD = 6.67, CI_95%_ = [4.07, 9.26]) and adults (*p* = 7.15 × 10^−7^; MD = 6.63, CI_95%_ = [4.15, 9.10]). Although across all age groups, accuracy to go trials was higher in the inhibition compared to vigilance condition (*p* = 9.17 × 10^−3^; MD = 1.55, CI_95%_ = [0.39, 2.71]), post‐hoc tests examining the age group‐by‐condition interaction revealed that only the children showed this effect (*p* = 5.15 × 10^−4^; MD = 4.05, CI_95%_ = [1.82, 6.29]). Across age groups go trial accuracy was higher in the inhibition compared to vigilance condition to angry faces (*p* = 1.79 × 10^−3^; MD = 2.39, CI_95%_ = [0.92, 3.87]); there was no effect of condition on happy faces (*p* = .27; MD = 0.71, CI_95%_ = [−0.57, 1.99]). There were no significant effects of emotion (*F*(2,95) = 1.15, *p* = .29; *η*_*p*_^2^ = 0.012), nor age group‐by‐emotion (*F*(2,94) = 0.97, *p* = .38; *η*_*p*_^2^ = 0.020) or age group‐by‐condition‐by‐emotion (*F*(2,94) = 2.05, *p* = .13; *η*_*p*_^2^ = 0.042) interactions on accuracy of go trials.

There were main effects of age group (*F*(2,94) = 8.57, *p* = 3.82 × 10^−4^; *η*_*p*_^2^ = 0.154) and condition (*F*(1, 115) = 731.78, *p* = 3.84 × 10^−46^; *η*_*p*_^2^ = 0.886), and age group‐by‐emotion (*F*(2,94) = 6.42, *p* = 2.44 × 10^−3^; *η*_*p*_^2^ = 0.120) and age group‐by‐condition‐by‐emotion (*F*(2,94) = 4.06, *p* = .02; *η*_*p*_^2^ = 0.079) interactions on the accuracy of the *no‐go trials* (Figure 1c; Table 2). Post‐hoc tests showed that accuracy was higher in the vigilance compared to inhibition condition (*p* = 3.84 × 10^−46^; MD = 20.83, CI_95%_ = [19.30, 22.36]). Children had significantly worse accuracy to no‐go trials across conditions and emotions compared to the adolescents (*p* = .02; MD = 3.31, CI_95%_ = [0.59, 6.03]) and adults (*p* = 7.59 × 10^−5^; MD = 5.42, CI_95%_ = [2.82, 8.02]). Over both conditions, the children showed significantly better no‐go accuracy to happy faces (*p* = .03; MD = 1.98, CI_95%_ = [1.5, 3.81]), while the adults had better accuracy to angry faces (*p* = .02; MD = 1.71, CI_95%_ = [0.26, 3.16]); the adolescents (*p* = .08; MD = 1.44, CI_95%_ = [−0.18, 3.06]) showed no effects. However, with respect to the three‐way interaction, this effect was seen in the vigilance condition for children (*p* = .02; MD = 1.29, CI_95%_ = [0.17, 2.41]) and the inhibition condition for the adults (*p* = .01; MD = 3.27, CI_95%_ = [0.66, 5.88]). There were no significant effects of emotion (*F*(2,95) = 1.42, *p* = .24; *η*_*p*_^2^ = 0.015), age group‐by‐condition (*F*(2,94) = 0.89, *p* = .42; *η*_*p*_^2^ = 0.019) or condition‐by‐emotion (*F*(2,95) = 0.01, *p* = .92; *η*_*p*_^2^ < 0.001) interactions on no‐go accuracy.

### 3.3 MEG results: age group‐by‐condition interactions

**FIGURE 2:**
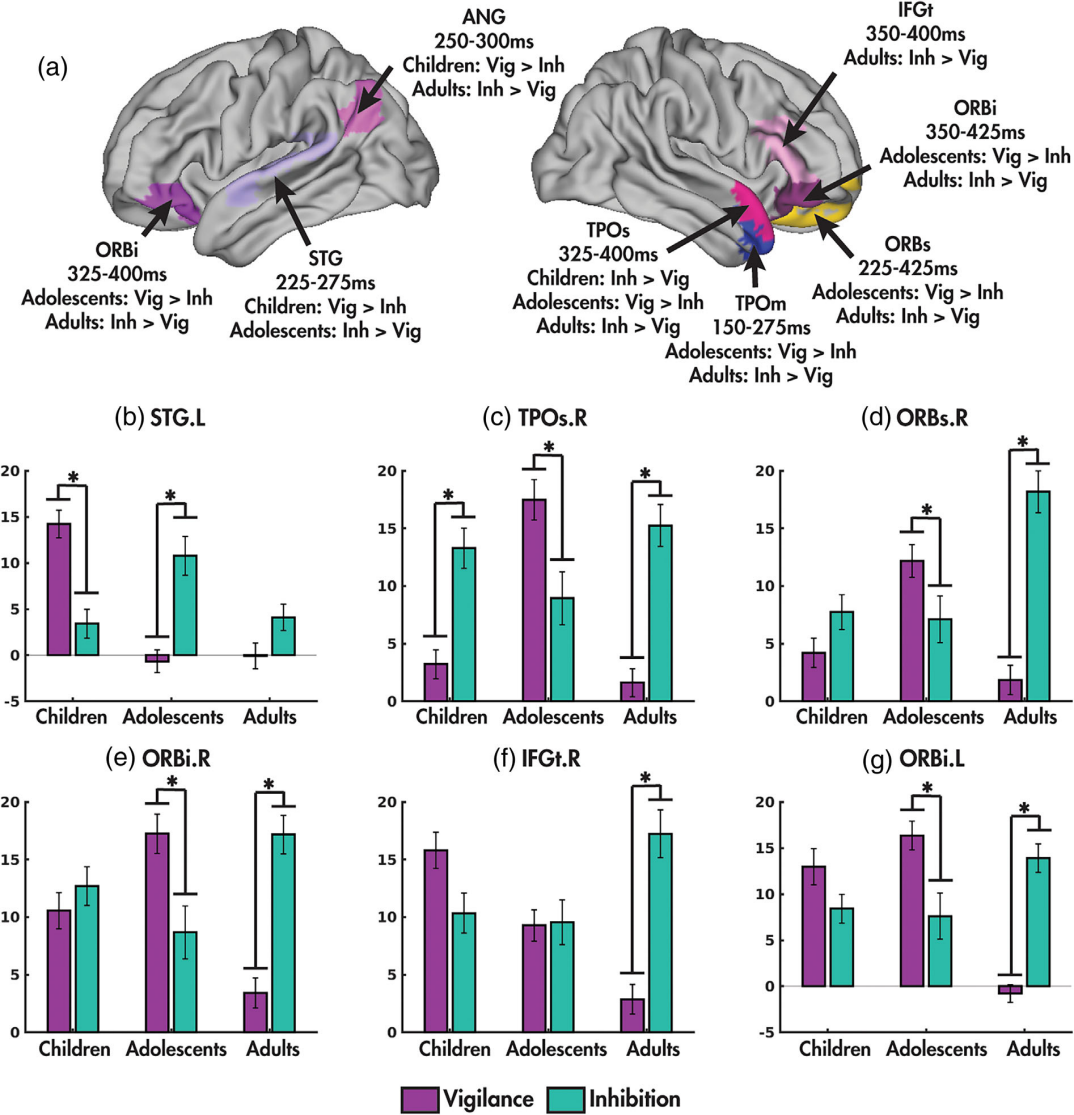
Age group‐by‐condition interactions. Regions showing significant age group‐by‐condition interactions are highlighted (a). The means and standard errors of power in each age group (children, adolescents and adults) for each condition (vigilance: purple, inhibition: green) are shown for the left superior temporal gyrus (b), right temporal pole (c), the right orbital parts of the superior (d) and inferior frontal (e) gyri, the triangular part of the right inferior frontal gyrus (f), and the left orbital inferior frontal gyrus (g). Significant post‐hoc tests (*p* < .05) indicated by asterisks

Age group‐by‐condition interactions were observed in subcortical, temporal and inferior frontal regions (Table 3A; Figure 2a). The subcortical regions included the right putamen (150–200 ms), left pallidum (200–275 ms) and thalamus (225–275 ms), and were driven by increased power during inhibition compared to vigilance in the adults, with the children showing the opposite effect in the putamen and thalamus. In the left superior temporal gyrus, between‐condition power differences were observed in the children and adolescents (225–275 ms), with the children showing increased power during vigilance and the adolescents during inhibition (Figure 2b). In the right middle temporal pole (150–275 ms), the adolescents demonstrated increased power during vigilance, while the adults did during inhibition trials. During a later window (325–400 ms) in the right superior temporal pole, both the children and the adults had increased power during inhibition, while the adolescents again had increased power during vigilance (Figure 2c). In the right orbital frontal gyrus ([ORBs] 225–425 ms; Figure 2d), and the right orbital ([ORBi] 350–425 ms; Figure 2e) and triangular ([IFGt] 350–425 ms; Figure 2f) parts of the IFG and left orbital IFG (325–400 ms; Figure 2g), adults demonstrated increased power during inhibition compared to vigilance; in all but the right triangular IFG, adolescents showed the opposite effect. The angular gyrus also showed age group‐by‐condition effects; however, due to the presence of an age group‐by‐condition‐by‐emotion interaction, results are discussed in the section below. Full timeseries for each significant region in the age groups are presented in Figure S1.

**TABLE 3:** Statistics related to the AAL regions showing significant (*p*_corr_ < .05) interactions with age

	AAL region	Time span of significance (ms)	*F*‐statistic	*p*_corr_	Post‐hocs (for *p*‐values see Table S4)					
					Children		Adolescents		Adults	
					V > I	I > V	V > I	I > V	V > I	I > V
**A. Age group‐by‐condition interactions**										
Frontal	ORBs.R	225–425	9.770	0.006			x			x
	ORBi.L	325–400	11.159	0.004			x			x
	ORBi.R	325–400	9.893	0.006			x			x
	IFGt.R	350–425	7.632	0.026						x
Temporal	TPOm.R	150–275	7.689	0.037			x			x
	STG.L	200–275	8.210	0.016	x					x
	TPOs.R	325–400	11.630	0.003		x	x			x
Parietal	ANG.L	250–300	7.620	0.039	x					x
Subcortical	PUT.R	150–200	8.403	0.037	x					x
	PAL.L	200–275	10.233	0.009						x
	THA.L	200–275	9.858	0.006	x					x

					Children		Adolescents		Adults	
					H > A	A > H	H > A	A > H	H > A	A > H
B. Age group‐by‐emotion interactions					Children		Adolescents		Adults	
Frontal	ORBs.L	250–300	8.184	0.048		x		x	x	
	ROL.R	350–400	8.660	0.032		x	x			
C. Age group‐by‐condition‐by‐emotion interactions					Children		Adolescents		Adults	
Parietal	ANG.L	250–300	11.508	0.003	V,H > I,H		I,H > V,H		I,A > V,A	

*Note*: The time window corresponds to the onset and offset of the aggregated significant time windows, and the *F*‐statistic and *p*_corr_‐values are reported for the time window with the maximum *F*‐statistic. Results of the post‐hoc tests are provided to show the directionality of effects.

Abbreviations: A, angry; AAL, Automated Anatomical Labelling atlas; ANG, angular gyrus; H, happy; I, inhibition; IFGt, triangular part of the inferior frontal gyrus; L, left; ORBs, orbital part of the superior orbital gyrus; ORBi, orbital part of the inferior frontal gyrus; PAL, pallidum; PUT, putamen; R, right; ROL, rolandic operculum; STG, superior temporal gyrus; TPOs, superior temporal pole; TPOm, middle temporal pole; THA, thalamus; V, vigilance.

### 3.4 MEG results: age group‐by‐emotion interactions

**FIGURE 3:**
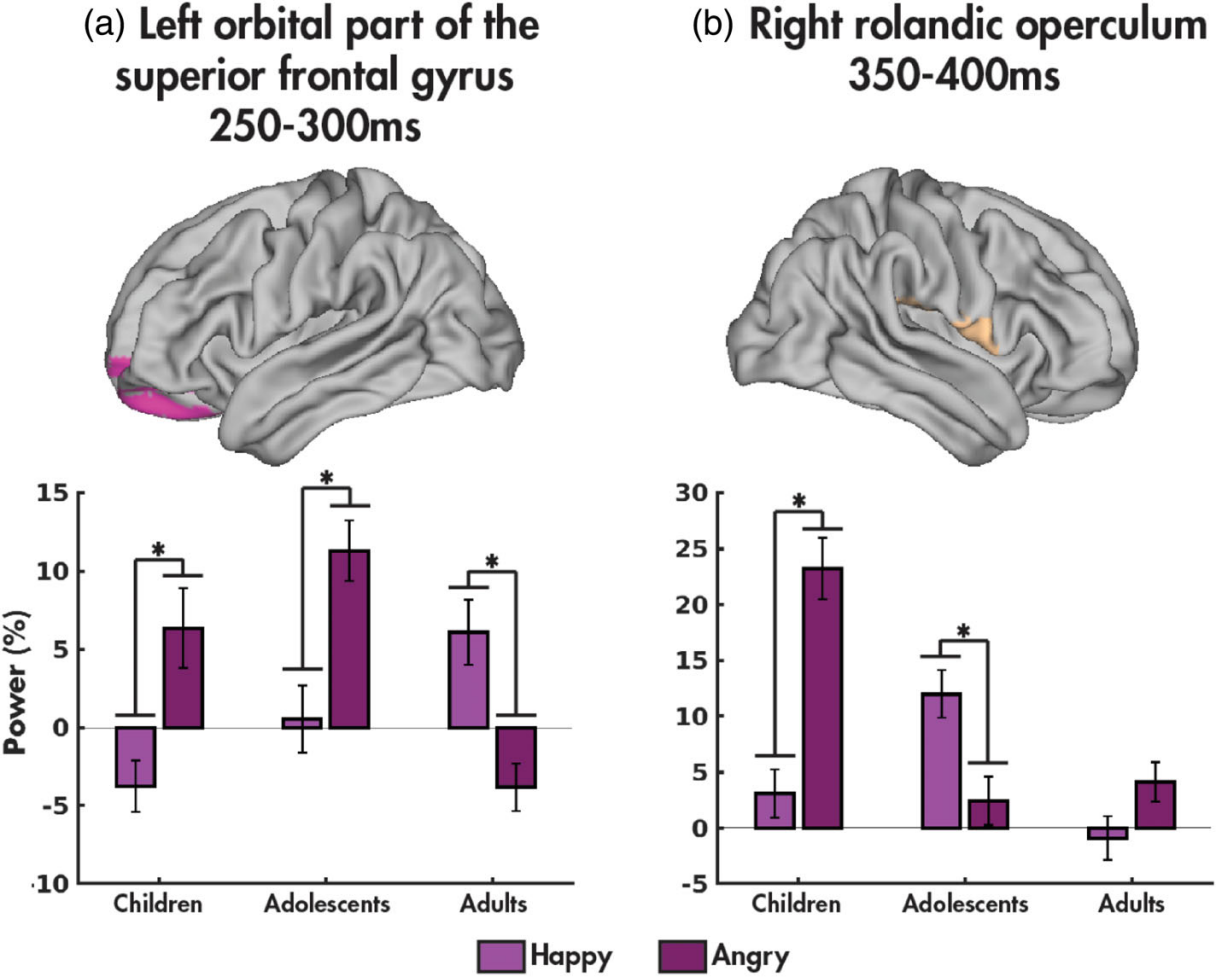
Age group‐by‐emotion interactions. The means and standard errors of power in each age group (children, adolescents and adults) for each emotion (happy: light purple, angry: dark purple) are shown for the left orbital frontal gyrus (a) and the right frontal rolandic operculum (b), with significant post‐hoc results (*p* < .05) indicated by an asterisk

An age group‐by‐emotion interaction was found in the left orbital frontal gyrus between 250 and 300 ms (*F*(2,94) = 8.184, *p*_corr_ = .048; Figure 3a; Table 3B). Post‐hoc tests revealed that power was increased to angry compared to happy faces in the children (*p* = .039) and adolescents (*p* = .011) yet increased to happy faces in the adults (*p* = .014). An interaction was also found in the right rolandic operculum between 350 and 400 ms post‐stimulus onset (*F*(2,94) = 8.660, *p*_corr_ = .032; Figure 3b). Power was increased to angry compared to happy faces in the children (*p* = 4.368 × 10^−4^), with the opposite effect observed in the adolescents (*p* = .037). Full timeseries for each significant region in the age groups are presented in Figure S2.

### 3.5 MEG results: age group‐by‐condition‐by‐emotion interaction

**FIGURE 4:**
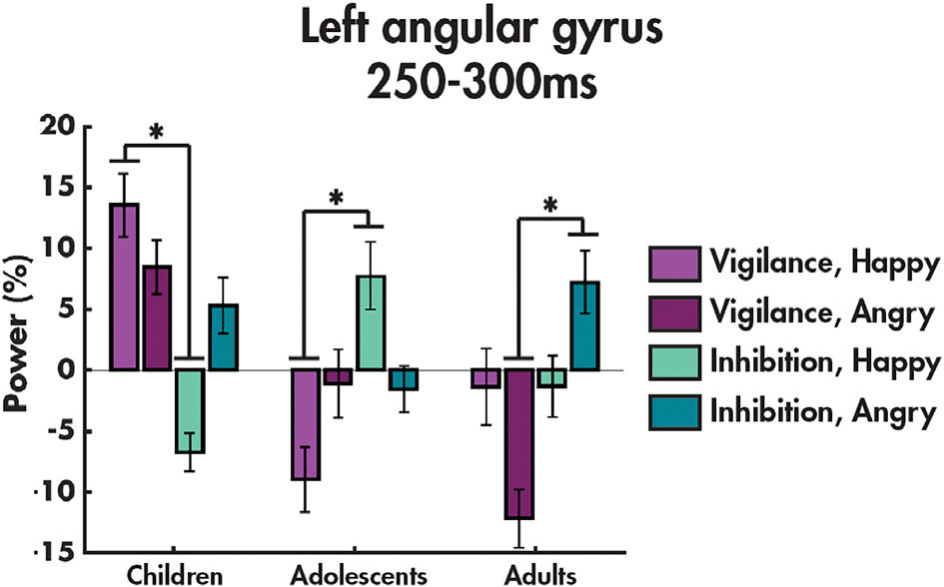
Age group‐by‐condition‐by‐emotion interaction. The means and standard errors of power in each age group (children, adolescents and adults) for each emotion are shown for angular gyrus, with significant post‐hoc results (*p* < .05) indicated by an asterisk

An age group‐by‐condition‐by‐emotion interaction was found in the left angular gyrus between 250 and 300 ms post stimulus onset (*F*(2,94) = 11.508, *p*_corr_ = .003; Figure 4; Table 3C). Post‐hoc tests showed that in this region, children exhibited increased power to happy faces in the vigilance compared to inhibition condition (*p* = .004), while adolescents showed the opposite effect (*p* = .009), and both groups showed no between‐condition differences to angry faces. In contrast, the adults only showed increased power to angry faces in the inhibition compared to vigilance condition (*p* = 2.99 × 10^−4^). Full timeseries for the significant region in the age groups are presented in Figure S3.

## 4 DISCUSSION

We used MEG and an emotional go/no‐go task to identify how the spatial and temporal characteristics of automatic emotion regulation change from childhood, through adolescence and into mid‐adulthood. Happy and angry faces were presented while participants performed two task conditions: inhibition, which created a prepotent tendency to respond, and vigilance, where the same stimuli were presented but without requiring response inhibition. Age‐related changes were observed in task performance and neural measures, and these were dependent on condition and emotion. Contrasting the inhibition and vigilance conditions, age‐related changes were observed in regions classically associated with automatic emotion regulation, such as the right inferior frontal and orbitofrontal cortices, and in primarily right‐lateralized temporal regions. The age groups also demonstrated differing patterns of activation of the left orbital superior frontal gyrus as a function of emotion. Finally, an interaction between age group, condition and emotion was found in the left angular gyrus, a region implicated in emotion regulation and attention.

### 4.1 Performance measures

Across the children, adolescents and adults, go trial RTs were faster and more accurate in the inhibition than vigilance condition, as well documented in the literature (Helton, 2009; Helton et al., 2005; Helton, Weil, Middlemiss, & Sawers, 2010; Staub, Doignon‐Camus, Bacon, & Bonnefond, 2014; Taylor et al., 2018). Our results demonstrate that this phenomenon is present even at young ages. However, only the adult group was better at withholding responses to angry compared to happy faces during inhibition, consistent with previous reports (Albert et al., 2010; Taylor et al., 2018). As expected, children had slower RTs and reduced accuracies regardless of the condition or emotion compared to adolescents, who in turn had slower RTs and lower accuracies compared to adults, concordant with reports of decreases in RTs and increases in accuracy with increasing age when performing go/no‐go tasks (Cohen Kadosh, Heathcote, & Lau, 2014; Iida, Miyazaki, & Uchida, 2010; van der Meere & Stemerdink, 1999).

### 4.2 Age‐related changes in inhibition

Our MEG findings revealed that power was increased in the right triangular and bilateral orbital parts of the IFG during inhibition compared to vigilance in adults, a classic finding in the literature (Aron, Fletcher, Bullmore, Sahakian, & Robbins, 2003; Chikazoe, Konishi, Asari, Jimura, & Miyashita, 2007; Dolcos, Kragel, Wang, & McCarthy, 2006; Taylor et al., 2018). The right orbital frontal gyrus is a region important for automatic emotion regulation; our findings substantiate that adults preferentially engage right‐lateralized prefrontal regions during emotional regulation (e.g., Mauss et al., 2007; Ochsner & Gross, 2005; Ochsner & Gross, 2007). Although the OFG showed increased activity during inhibition compared to baseline in children, consistent with prior work (Urbain et al., 2017), the lack of significant effects in the OFG in the children in the current study, supports a model for the increasing recruitment of the prefrontal cortex with age during emotion regulation (Bunge, Dudukovic, Thomason, Vaidya, & Gabrieli, 2002; Lewis et al., 2006).

Interestingly, adolescents showed increased power in the orbitofrontal regions during vigilance compared to inhibition (right orbital and bilateral inferior frontal gyri). Like the children, power was still increased compared to baseline during inhibition, suggesting there is still involvement of these regions during inhibition as observed in the adults, but more so during vigilance. Along with its involvement in emotion regulation, the orbitofrontal cortex is important in the representation of rewards and punishments (Rolls, 2000; Rolls & Grabenhorst, 2008). Adolescence is a period with a heightened dependence on social cues, and adolescents are more likely to engage in risky behaviour to obtain rewards, particularly with respect to their peers (Albert, Chein, & Steinberg, 2013; Guyer, Choate, Pine, & Nelson, 2012; Somerville, Somerville, Dir, & Sci, 2013). Thus, when cognitive load is reduced during the easier vigilance task, the adolescents, an age where social cues are heavily relied upon, may be activating this reward region to determine the value of the facial expressions. This increased activation to vigilance trials in adolescents and inhibition trials in adults started early (225 ms) and continued until 425 ms, consistent with sustained involvement in the orbital frontal regions during emotional regulation tasks (Urbain et al., 2017), and extend these results to larger cohorts, and including adolescents. Slightly superior bilateral orbital regions showed the same patterns of activity in the adolescents and adults, but from 325 to 400 ms in the left and 350–425 in the right hemisphere, suggesting that more extensive processing occurs in the older two age groups in these regions.

In adults there was also an increase in activity at 350–400 ms in the IFG, a longer latency than the peak of typical IFG activity to inhibition, previously reported at 230–270 ms in adults and adolescents (Taylor et al., 2018; Vara et al., 2014). As age by condition effects were not seen, this suggests that this area is involved across the age range, including the children, in the earlier time windows. This protracted activation in adults could be understood as more extensive processing; the factors that contribute to this prolonged increase would be of interest for future studies.

Age group‐by‐condition interactions were also found in temporal regions: the left superior temporal gyrus and right temporal pole. Our results of early brief activation in the left superior temporal gyrus in children and adolescents, but not adults, points to an immaturity in the lateralization of these processes, as the right superior temporal gyrus is a key part of the emotional face processing system (Haxby et al., 2000), and was identified in a meta‐analysis examining regions involved in response inhibition go/no‐go tasks (Criaud & Boulinguez, 2013) in adults. The fact that there were no effects seen for the right STG indicates that this region was engaged similarly for emotion regulation across the three age groups; the left STG was engaged additionally only in the two younger groups.

The importance of the right temporal pole to this task is supported by our current findings of increased power during inhibition in children and adults. The adolescents, however, showed increased power during vigilance in the right anterior temporal lobe. It is hypothesised that the temporal poles are vital in linking communication between the inferior frontal and orbitofrontal gyri (Olson, Plotzker, & Ezzyat, 2007; Taylor et al., 2018) to enable correct responses to the emotional stimuli, and thus this effect in adolescence we suggest is a reflection of the increases in power during vigilance in inferior frontal and orbitofrontal regions. This increased activity in the medial temporal pole occurred very early (150–275 ms) in the adolescents (to vigilance trials) and adults (to inhibition trials) underscoring the importance of the temporal pole in rapid recognition of emotionally relevant stimuli. The temporal poles are often linked to the salience network (Doll, Hölzel, Boucard, Wohlschläger, & Sorg, 2015; Menon, 2011), a network critical in quick detection of salient stimuli. The superior temporal pole showed increased activation in all three age groups at a later time point (325–400 ms), consistent with the role of the temporal pole in higher level social cognitive processing (Olson et al., 2007). Our finding of only right temporal pole activity during this task aligns with evidence of the right temporal pole being associated with emotional stimuli and the left temporal pole being associated with semantic information (see Olson et al., 2007 for a review).

### 4.3 Age‐related changes in emotion processing

An interaction between age group and emotion was found in the left orbital frontal gyrus and the right frontal rolandic operculum. The orbital frontal brain is critical for emotional regulation, social cognitive function including reward and emotional processing (Bachevalier, Machado, & Kazama, 2011; Mundy, 2018; Rolls, 2019). Children and adolescents recruited this region more in response to angry compared to happy faces, while adults showed the opposite effect. An fMRI study with a similar protocol, also showed increasing activation with age of the left orbital frontal cortex to angry faces (Todd et al., 2012). They suggested that this increase reflected the dissonance between approach signalled by happy faces (Roelofs, Minelli, Mars, van Peer, & Toni, 2009) and the need to withhold a response, as this area has been linked to conflict management (Volman, Roelofs, Koch, Verhagen, & Toni, 2011). The increase to happy faces in adults could be due to the positivity bias, which is the tendency to favour positive over negative stimuli (Denkinger & Kinn, 2018), which increases over adulthood. Others have also reported increased activity in left ventromedial prefrontal cortex to positive emotional faces and reduced to negative faces in typical adults (Eack et al., 2016). The current data further reinforce the rapidity of the involvement of orbital frontal areas (250–300 ms) in processing emotional stimuli (e.g., Taylor et al., 2018; Urbain et al., 2017).

The frontal operculum has been strongly implicated in task control (Dosenbach et al., 2006; Higo, Mars, Boorman, Buch, & Rushworth, 2011), but not studied in emotional tasks across age. Our results suggest that children invoke greater effort during this task for the angry faces, likely due to less experience with angry faces. The adolescents may, again, be more focused on the reward value of happy faces, while the facial emotions did not affect the task control aspect in adults. Future studies are needed to delve further into this finding and determine age and task variables that influence task control, as indexed by frontal operculum activity.

### 4.4 Age‐dependent interactions in automatic emotion regulation

Only the left angular gyrus showed an age group‐by‐condition‐by‐emotion interaction from 250 to 300 ms. The angular gyri play crucial roles in emotion regulation (Kohn et al., 2014), as well as extending to a range of other cognitive functions, including the reorienting and shifting of attention (see Seghier, 2013, for a review). We found that adults activated this region more during inhibition compared to vigilance when viewing angry faces, consistent with a previous fMRI study that found increased recruitment of the bilateral angular gyri during inhibition to aversive compared to neutral stimuli in adults (Brown et al., 2012). These results suggest that in adults, successfully executing emotion regulation by inhibiting a response to angry faces induces increased attentional demands, yet during vigilance, this region is supressed, perhaps to enable the recruitment of core emotion processing regions. In contrast, children and adolescents do not show this increased power in the left angular gyrus to angry faces during inhibition. This could be an immature response and underlie the reduced accuracy in the no‐go trials in the younger participants compared to the adults that was specific to angry faces.

Nonlinear development was again seen with the adolescents, the only group who showed increased power in the left angular gyrus during inhibition to happy faces. A previous behavioural study reported that when performing a working memory task, happy but not angry faces, modulated performance in adolescents, but not adults, suggesting that adolescents alone are distracted by the happy faces, and that attentional resources are reallocated away from the task at hand to the positive stimuli (Cromheeke & Mueller, 2016). Adolescents appeared to require increased activation of this attention region to perform the task and to overcome their heightened propensity to attend, or seek out, positive or rewarding stimuli (Albert et al., 2013; Guyer et al., 2012; Somerville et al., 2013). This is consistent with our findings of increased activation of the orbitofrontal cortex, a reward region, during vigilance: when less attention is required to perform the task, the adolescents are evaluating the facial expressions for reward value. Finally, increased power in the left angular gyrus to happy faces in the vigilance compared to inhibition condition in children may reflect immature emotion regulation, although the preference towards happy faces is already present in childhood.

A limitation to the study is the use of age bins, rather than using age as a continuous variable. Other studies have also shown nonlinear development, particularly with poorer performance in adolescence (Cohen‐Gilbert & Thomas, 2013; Lewis et al., 2006), and hence leading to studies that focus on adolescents (Ahmed et al., 2015; Schweizer et al., 2020; Zhang et al., 2016). Our work reinforces these nonlinear effects in brain function related to automatic emotion regulation and suggests that future work should examine specifically the nature of these nonlinearities, not only across the wide age range used here but also within narrower age groupings. Another potential confound of this study is the use of variable stimuli duration to maintain steady error rates (see Table S2); although this task design decision was necessary to ensure that all participants could complete the task, and longer durations presented to the children coincides with the protracted neuronal responses to faces in younger populations, these differences may contribute to the discussed effects.

## 5 CONCLUSIONS

In conclusion, using MEG we determined the developmental changes from young childhood to adulthood in the neural mechanisms supporting automatic emotion regulation via an emotional go/no‐go task. Age‐related changes were observed in both task performance and neural measures, and these were dependent on condition and emotion. Our findings demonstrate protracted development of automatic emotional processing from child to adulthood which are modulated by emotional face type, and highlight the marked nonlinear effects during adolescence. We also established the rapid interplay in the first 150–425 ms after stimulus presentation of key frontal, temporal and parietal regions involved in this complex social cognitive process of automatic emotion regulation, and how they evolve with age. Our study lays the framework for future investigations into how clinical populations may deviate from the normative development of emotional regulation.

## CONFLICT OF INTEREST

E. Anagnostou has served as a consultant to Roche and Quadrant Therapeutics, holds a patent for the device, ‘Anxiety Meter’, and has received in‐kind support from AMO Pharma, royalties from APPI and Springer, and editorial honoraria from Wiley. The remaining authors (M. M. Vandewouw, K. Safar, J. Sato, B. A. E. Hunt, C. M. Urbain, and M. J. Taylor) have no competing interests to declare.

## ETHICS APPROVAL

This research was approved by the Hospital for Sick Children Research Ethics Board and conforms with the Declaration of Helsinki. Informed consent was received by participants who were old enough to do so; otherwise, informed assent was obtained from the participant and informed written consent from the caregiver.

## Supporting information


**Appendix** **S1:** Supplementary Information

## Data Availability

The data that support the findings of this study are available from the corresponding author upon reasonable request.
