# Supplementary material for: Ignore the faces: Neural characterisation of emotional inhibition from childhood to adulthood using MEG
Source: Hum Brain Mapp. 2021 Sep 28;42(17):5747–60. doi: 10.1002/hbm.25651 (PMC8559465; doi:10.1002/hbm.25651)
Supplement: Supplementary file 1 — Appendix S1: Supplementary Information [file HBM-42-5747-s001.docx]

**Ignore the faces: Neural characterization of emotional inhibition from childhood to adulthood using MEG**

Marlee M. Vandewouw, Kristina Safar, Julie Sato, Benjamin A.E. Hunt, Charline M. Urbain, Elizabeth W. Pang, Evdokia Anagnostou, Margot J. Taylor

**Supporting Information**

**Supplemental Table 1:** Regions from the AAL atlas, their abbreviations in centroids in MNI space.

| **AAL region** | **Abbreviation** | **Centroid MNI coordinates** | | |
| --- | --- | --- | --- | --- |
|  |  | **X (mm)** | **Y (mm)** | **Z (mm)** |
| Precental gyrus | PreCG.L | -40 | -6 | 51 |
|  | PreCG.R | 40 | -8 | 52 |
| Superior frontal gyrus, dorsolateral | SFGd.L | -19 | 35 | 42 |
|  | SFGd.R | 20 | 31 | 44 |
| Superior frontal gyrus, orbital part | ORBs.L | -18 | 47 | -13 |
|  | ORBs.R | 17 | 48 | -14 |
| Middle frontal gyrus | MFG.L | -34 | 33 | 35 |
|  | MFG.R | 37 | 33 | 34 |
| Middle frontal gyrus, orbital part | ORBm.L | -32 | 50 | -10 |
|  | ORBm.R | 32 | 53 | -11 |
| Inferior frontal gyrus, opercular part | IFGo.L | -49 | 13 | 19 |
|  | IFGo.R | 49 | 15 | 21 |
| Inferior frontal gyrus, triangular part | IFGt.L | -47 | 30 | 14 |
|  | IFGt.R | 49 | 30 | 14 |
| Inferior frontal gyrus, orbital part | ORBi.L | -37 | 31 | -12 |
|  | ORBi.R | 40 | 32 | -12 |
| Rolandic operculum | ROL.L | -48 | -8 | 14 |
|  | ROL.R | 52 | -6 | 15 |
| Supplementary motor area | SMA.L | -6 | 5 | 61 |
|  | SMA.R | 8 | 0 | 62 |
| Olfactory cortex | OLF.L | -9 | 15 | -12 |
|  | OLF.R | 8 | 16 | -11 |
| Superior frontal gyrus, medial | SFGm.L | -6 | 49 | 31 |
|  | SFGm.R | 8 | 51 | 30 |
| Superior frontal gyrus, medial orbital | ORBsm.L | -6 | 54 | -7 |
|  | ORBsm.R | 7 | 52 | -7 |
| Gyrus rectus | REC.L | -6 | 37 | -18 |
|  | REC.R | 7 | 36 | -18 |
| Insula | INS.L | -36 | 7 | 3 |
|  | INS.R | 38 | 6 | 2 |
| Anterior cingulate and paracingulate gyri | ACG.L | -5 | 35 | 14 |
|  | ACG.R | 7 | 37 | 16 |
| Median cingulate and paracingulate gyri | DCG.L | -6 | -15 | 42 |
|  | DCG.R | 7 | -9 | 40 |
| Posterior cingulate gyrus | PCG.L | -6 | -43 | 25 |
|  | PCG.R | 6 | -42 | 22 |
| Hippocampus | HIP.L | -26 | -21 | -10 |
|  | HIP.R | 28 | -20 | -10 |
| Parahippocampal gyrus | PHG.L | -22 | -16 | -21 |
|  | PHG.R | 24 | -15 | -20 |
| Amygdala | AMYG.L | -24 | -1 | -17 |
|  | AMYG.R | 26 | 1 | -18 |
| Calcarine fissure and surrounding cortex | CAL.L | -8 | -79 | 6 |
|  | CAL.R | 15 | -73 | 9 |
| Cuneus | CUN.L | -7 | -80 | 27 |
|  | CUN.R | 13 | -79 | 28 |
| Lingual gyrus | LING.L | -16 | -68 | -5 |
|  | LING.R | 15 | -67 | -4 |
| Superior occipital gyrus | SOG.L | -18 | -84 | 28 |
|  | SOG.R | 23 | -81 | 31 |
| Middle occipital gyrus | MOG.L | -33 | -81 | 16 |
|  | MOG.R | 36 | -80 | 19 |
| Inferior occipital gyrus | IOG.L | -37 | -78 | -8 |
|  | IOG.R | 37 | -82 | -8 |
| Fusiform gyrus | FFG.L | -32 | -40 | -20 |
|  | FFG.R | 33 | -39 | -20 |
| Postcentral gyrus | PoCG.L | -43 | -23 | 49 |
|  | PoCG.R | 40 | -25 | 53 |
| Superior parietal gyrus | SPG.L | -24 | -60 | 59 |
|  | SPG.R | 25 | -59 | 62 |
| Inferior parietal, but supramarginal and angular gyri | IPL.L | -44 | -46 | 47 |
|  | IPL.R | 45 | -46 | 50 |
| Supramarginal gyrus | SMG.L | -57 | -34 | 30 |
|  | SMG.R | 57 | -32 | 34 |
| Angular gyrus | ANG.L | -45 | -61 | 36 |
|  | ANG.R | 45 | -60 | 39 |
| Precuneus | PCUN.L | -8 | -56 | 48 |
|  | PCUN.R | 9 | -56 | 44 |
| Paracentral lobule | PCL.L | -9 | -25 | 70 |
|  | PCL.R | 6 | -32 | 68 |
| Caudate nucleus | CAU.L | -12 | 11 | 9 |
|  | CAU.R | 14 | 12 | 9 |
| Lenticular nucleus, putamen | PUT.L | -25 | 4 | 2 |
|  | PUT.R | 27 | 5 | 2 |
| Lenticular nucleus, pallidum | PAL.L | -19 | 0 | 0 |
|  | PAL.R | 20 | 0 | 0 |
| Thalamus | THA.L | -12 | -18 | 8 |
|  | THA.R | 12 | -18 | 8 |
| Heschl gyrus | HES.L | -43 | -19 | 10 |
|  | HES.R | 45 | -17 | 10 |
| Superior temporal gyrus | STG.L | -54 | -21 | 7 |
|  | STG.R | 57 | -22 | 7 |
| Temporal pole: superior temporal gyrus | TPOs.L | -41 | 15 | -20 |
|  | TPOs.R | 47 | 15 | -17 |
| Middle temporal gyrus | MTG.L | -57 | -34 | -2 |
|  | MTG.R | 56 | -37 | -1 |
| Temporal pole: middle temporal gyrus | TPOm.L | -37 | 15 | -34 |
|  | TPOm.R | 43 | 15 | -32 |
| Inferior temporal gyrus | ITG.L | -51 | -28 | -23 |
|  | ITG.R | 53 | -31 | -22 |

**Supplemental Table 2:** Descriptive statistics of the stimulus duration for both conditions (INH: inhibition, VIG: vigilance) and emotion (H: happy, A: angry) in the three age groups.

|  | | **Children**  **(4–10 years)** | **Adolescents**  **(11–19 years)** | **Adults**  **(20–40 years)** |
| --- | --- | --- | --- | --- |
| **Mean stimulus duration**  **(ms ± std.)** | **VIG, H** | 483 ± 84 | 393 ± 33 | 347 ± 32 |
|  | **VIG, A** | 485 ± 82 | 388 ± 29 | 347 ± 31 |
|  | **INH, H** | 635 ± 75 | 563 ± 81 | 417 ± 50 |
|  | **INH, A** | 638 ± 76 | 562 ± 81 | 418 ± 51 |

VIG: vigilance, INH: inhibition, H: happy, A: angry

There were main effects of age group (*F*(2,94)=114.50, *p*=6.38×10-26) and condition (*F*(2,95)=303.15, *p*=3.61×10-31), and an age group-by-condition (*F*(2,94)=19.30, *p*=9.51×10-8) interaction on the stimulus duration of the no-go trials. Post-hoc tests found that, consistent with expected developmental trends, the children had significantly longer stimulus durations compared to both the adolescents (*p*=5.25×10-9) and adults (*p*=9.56×10-10), and adolescents had longer durations compared to adults (*p*=9.56×10-10). Across all age groups, duration was longer in the inhibition compared to vigilance condition (*p*=1.06×10-10) consistent with the increased difficulty of the inhibition task. Furthermore, the effect was less dramatic in the adults (*p*=2.17×10-8) who had a mean difference (MD) of 71ms, than in the children (MD=153.0ms, *p*=1.06×10-10) and adolescents (MD=172ms, *p*=1.06×10-10). There were no significant effects of emotion (*F*(2,95)= 4.32×10-4, *p*=0.98), nor age group-by-emotion (*F*(2,94)=2.23, *p*=0.11), condition-by-emotion (*F*(2,95)=0.77, *p*=0.38) or age group-by-condition-by-emotion (*F*(2,94)=0.33, *p*=0.72) interactions on stimulus duration.

**Supplemental Table 3:** Summary of statistics relating to the task performance measures, including full listing of post-hoc *p*-value results for significant omnibus tests. Post-hoc tests were only run upon significance of the omnibus, and thus are not included for non-significant effects. The directionality of the post-hoc test is indicated by its corresponding column, and omitted for the opposite direction.

| **Main effect of age group** | | | **Post-hoc *p*-values (only run upon significance)** | | | | | | | | | | | | | | |
| --- | --- | --- | --- | --- | --- | --- | --- | --- | --- | --- | --- | --- | --- | --- | --- | --- | --- |
|  | ***F*-statistic** | ***p*_corr_** | **C > Ad** | | **Ad > C** | | | **C > AD** | | | **AD > C** | | **Ad > AD** | | | **AD > Ad** | |
| **Reaction time** | 73.34 | 4.36×10^-20^ | 3×10^-15^ | | - | | | 1×10^-32^ | | | - | | 0.04 | | | - | |
| **Go accuracy** | 17.13 | 4.54×10^-7^ | - | | 1.7×10^-6^ | | | - | | | 7.1×10^-7^ | | - | | | 0.97 | |
| **No-go accuracy** | 8.57 | 3.8×10^-4^ | - | | 0.02 | | | - | | | 7.6×10^-5^ | | - | | | 0.09 | |
| **Main effect of condition** | | | **Post-hoc tests** | | | | | | | | | | | | | | |
|  | ***F*-statistic** | ***p*_corr_** | **V > I** | | | | | | | | **I > V** | | | | | | |
| **Reaction time** | 14.47 | 2.53×10^-4^ | 2.5×10^-4^ | | | | | | | | - | | | | | | |
| **Go accuracy** | 7.08 | 0.01 | - | | | | | | | | 0.01 | | | | | | |
| **No-go accuracy** | 731.78 | 3.84×10^-46^ | 3.8×10^-46^ | | | | | | | |  | | | | | | |
| **Main effect of emotion** | | | **Post-hoc tests** | | | | | | | | | | | | | | |
|  | ***F*-statistic** | ***p*_corr_** | **H > A** | | | | | | | | **A > H** | | | | | | |
| **Reaction time** | 1.42 | 0.24 | - | | | | | | | | - | | | | | | |
| **Go accuracy** | 1.15 | 0.29 | - | | | | | | | | - | | | | | | |
| **No-go accuracy** | 1.42 | 0.24 | - | | | | | | | | - | | | | | | |
| **Age group × condition** | | | **Post-hoc tests** | | | | | | | | | | | | | | |
|  | ***F*- statistic** | ***p*_corr_** | **Children** | | | | | | **Adolescents** | | | | | **Adults** | | | |
|  |  |  | **V > I** | | **I > V** | | | **V > I** | | | **I > V** | | **V > I** | | | **I > V** | |
| **Reaction time** | 2.70 | 0.07 | - | | - | | | - | | | - | | - | | | - | |
| **Go accuracy** | 4.67 | 0.01 | - | | 5.1×10^-4^ | | | - | | | 0.37 | | 0.74 | | | - | |
| **No-go accuracy** | 0.89 | 0.42 | - | | - | | | - | | | - | | - | | | - | |
| **Age group × emotion** | | | **Post-hoc tests** | | | | | | | | | | | | | | |
|  | ***F*- statistic** | ***p*_corr_** | **Children** | | | | | | **Adolescents** | | | | | **Adults** | | | |
|  |  |  | **H > A** | | **A > H** | | | **H > A** | | | **A > H** | | **H > A** | | | **A > H** | |
| **Reaction time** | 0.99 | 0.38 | - | | - | | | - | | | - | | - | | | - | |
| **Go accuracy** | 0.97 | 0.38 | - | | - | | | - | | | - | | - | | | - | |
| **No-go accuracy** | 6.42 | 2.4×10^-3^ | 0.03 | | - | | | 0.08 | | | - | | 0.02 | | | - | |
| **Condition × emotion** | | | **Post-hoc tests** | | | | | | | | | | | | | | |
|  | ***F*- statistic** | ***p*_corr_** | **H** | | | | | | | | **A** | | | | | | |
|  |  |  | **V > I** | | | | **I > V** | | | | **V > I** | | | | **I > V** | | |
| **Reaction time** | 2.86 | 0.09 | - | | | | - | | | | - | | | | - | | |
| **Go accuracy** | 4.90 | 0.03 | - | | | | 0.3 | | | | - | | | | 1.8×10^-3^ | | |
| **No-go accuracy** | 0.01 | 0.92 | - | | | | - | | | | - | | | | - | | |
| **Age group × emotion × condition** | | | **Children** | | | | | | **Adolescents** | | | | | **Adults** | | | |
|  | ***F*- statistic** | ***p*_corr_** | **H > A** | | **A > H** | | | **H > A** | | | **A > H** | | **H > A** | | | **A > H** | |
|  |  |  | **V** | **I** | **V** | **I** | | **V** | | **I** | **V** | **I** | **V** | | **I** | **V** | **I** |
| **Reaction time** | 2.04 | 0.14 | - | - | - | - | | - | | - | - | - | - | | - | - | - |
| **Go accuracy** | 2.05 | 0.13 | - | - | - | - | | - | | - | - | - | - | | - | - | - |
| **No-go accuracy** | 4.06 | 0.02 | 0.02 | 0.11 | - | - | | 0.39 | | 0.10 | - | - | - | |  | 0.74 | 0.01 |

C: children, Ad: adolescents, AD: adults, V: vigilance, I: inhibition, H: happy, A: angry

**Supplemental Table 4:** Post-hoc *p*-values for regions showing significant (*p*_corr_ < 0.05) interactions with age as presented in Table 2 in the main text.

|  | **AAL region** | **Post-hocs**  **(for *p*-values see Supplemental Table 2)** | | | | | |
| --- | --- | --- | --- | --- | --- | --- | --- |
| 1. **Age group-by-condition interactions** | | **Children** | | **Adolescents** | | **Adults** | |
|  |  | **V > I** | **I > V** | **V > I** | **I > V** | **V > I** | **I > V** |
| Subcortical | PUT.R | 0.002 |  | 0.938 |  |  | 0.014 |
|  | PAL.L | 0.075 |  | 0.315 |  |  | 5.755×10^-5^ |
|  | THA.L | 0.002 |  |  | 0.298 |  | 0.003 |
| Temporal | STG.L | 0.009 |  |  | 0.004 |  | 0.197 |
|  | TPOs.R |  | 0.019 | 0.013 |  |  | 8.511×10^-5^ |
|  | TPOm.R | 0.170 |  | 0.013 |  |  | 0.008 |
| Parietal | ANG.L | 0.011 |  |  | 0.066 |  | 0.009 |
| Frontal | ORBs.R |  | 0.183 | 0.019 |  |  | 1.597×10^-4^ |
|  | IFGt.R | 0.099 |  |  | 0.477 |  | 8.566×10^-5^ |
|  | ORBi.L | 0.175 |  | 0.014 |  |  | 2.608×10^-4^ |
|  | ORBi.R |  | 0.464 | 0.015 |  |  | 1.680×10^-4^ |
|  | OLF.R |  | 0.259 | 0.032 |  |  | 3.076×10^-4^ |
| 1. **Age group-by-emotion interactions** | | **Children** | | **Adolescents** | | **Adults** | |
|  |  | **H > A** | **A > H** | **H > A** | **A > H** | **H > A** | **A > H** |
| Frontal | ORBs.L |  | 0.039 |  | 0.011 | 0.014 |  |
|  | ROL.R |  | 4.368×10^-4^ | 0.037 |  |  | 0.242 |
| 1. **Age group-by-condition-by-emotion interactions** | | **Children** | | **Adolescents** | | **Adults** | |
| Parietal | ANG.L | I,A > I,H: 0.107  V,A > I,A: 0.928  V,H > I,A: 0.547  V,A > I,H: 0.084  V,H > I,H: 0.004  V,H > V,A: 0.855 | | I,H > I,A: 0.200  V,A > I, A: 0.995  I,A > V,H: 0.566  I,H > V,A: 0.511  I,H > V,H: 0.009  V,A > V,H: 0.384 | | I,A > I,H: 0.173  I,A > V,A: 2.99×10^-4^  I,A > V,H: 0.314  I,H > V,A: 0.154  I,H > V,H: 1.000  V.H > V,A: 0.107 | |

AAL: Automated Anatomical Labeling atlas, V: vigilance, I: inhibition, H: happy, A: angry, R: right, L: left, ORBs: orbital part of the superior orbital gyrus, IFGt: triangular part of the inferior frontal gyrus, ORBi: orbital part of the inferior frontal gyrus, STG: superior temporal gyrus, TPOs: superior temporal pole, TPOm: middle temporal pole, ANG: angular gyrus, PUT: putamen, PAL: pallidum, THA: thalamus, ROL: rolandic operculum

**
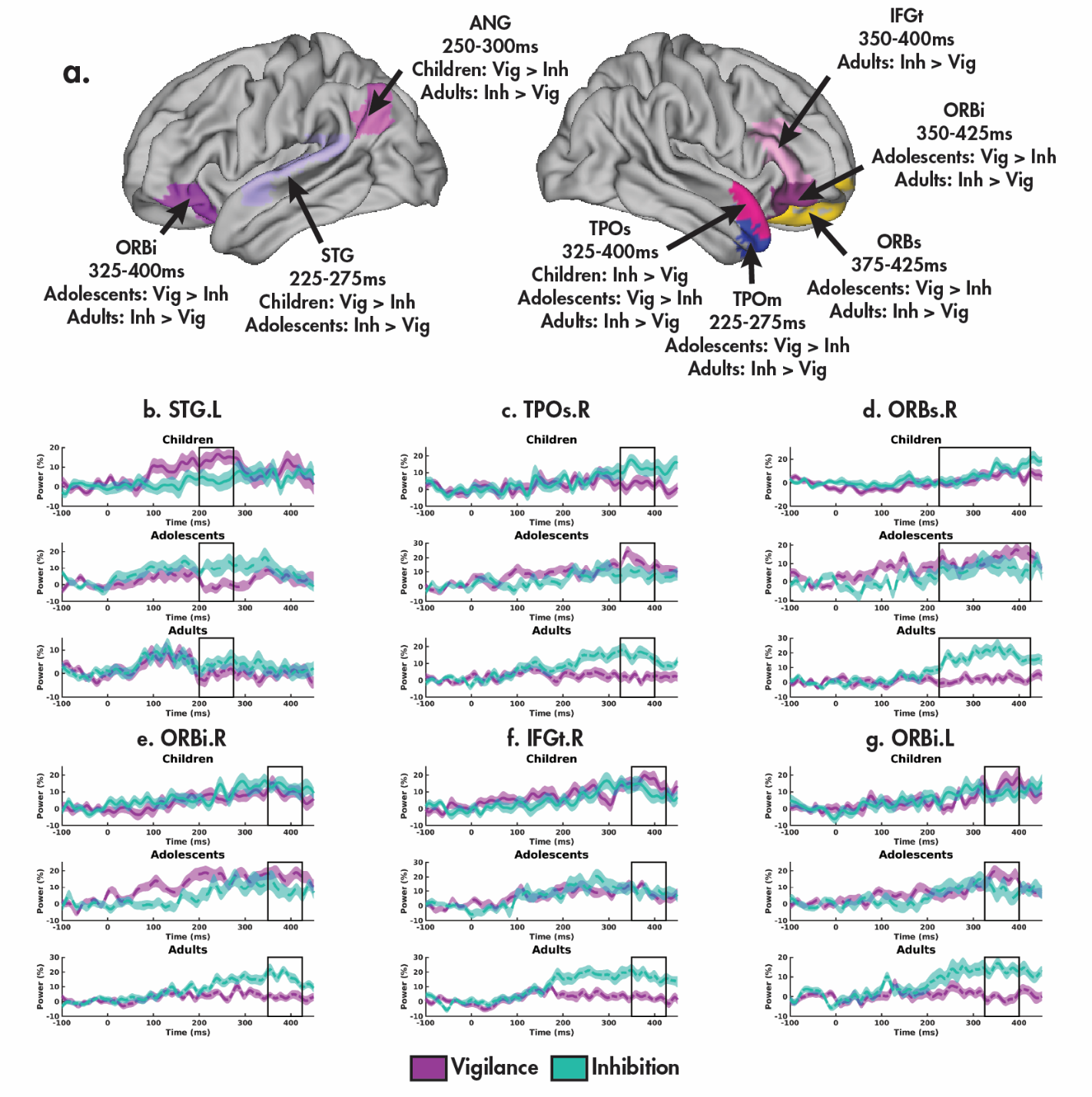
**

**Supplemental Figure 1:** Age group-by-condition interactions. Regions showing significant age group-by-condition interactions are highlighted (a). The means and standard errors of the power timeseries in each age group (children, adolescents and adults) for each condition (vigilance: purple, inhibition: green) are shown for the left superior (b) temporal gyrus, right temporal pole (c), the right orbital parts of the superior (d) and inferior frontal (e) gyri, the triangular part of the right inferior frontal gyrus (f), and the left orbital inferior frontal gyrus (g).


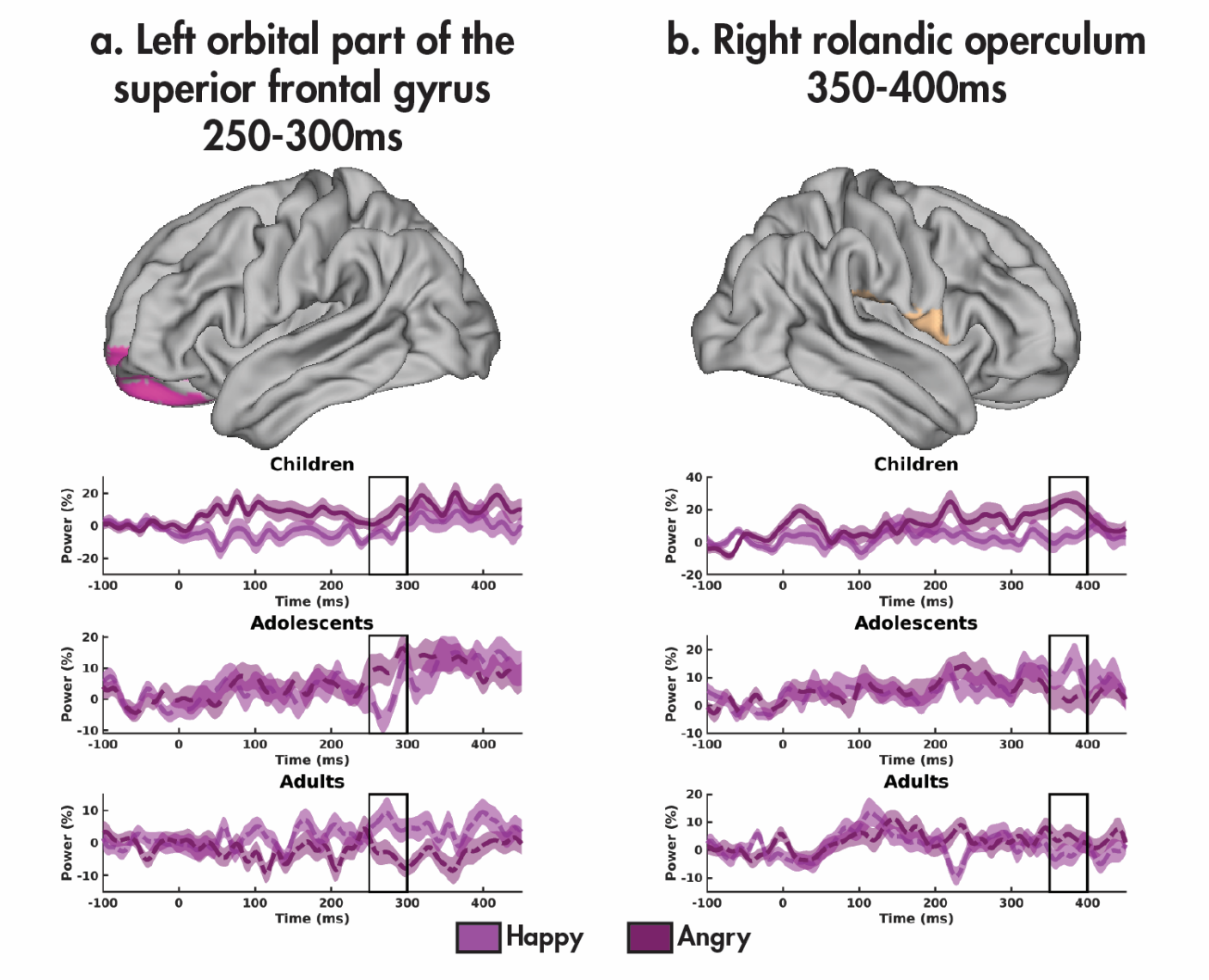


**Supplemental Figure 2:** Age group-by-emotion interactions. The means and standard errors of power timeseries in each age group (children, adolescents and adults) for each emotion (happy: light purple, angry: dark purple) are shown for the left orbital frontal gyrus (a) and the right frontal rolandic operculum (b).


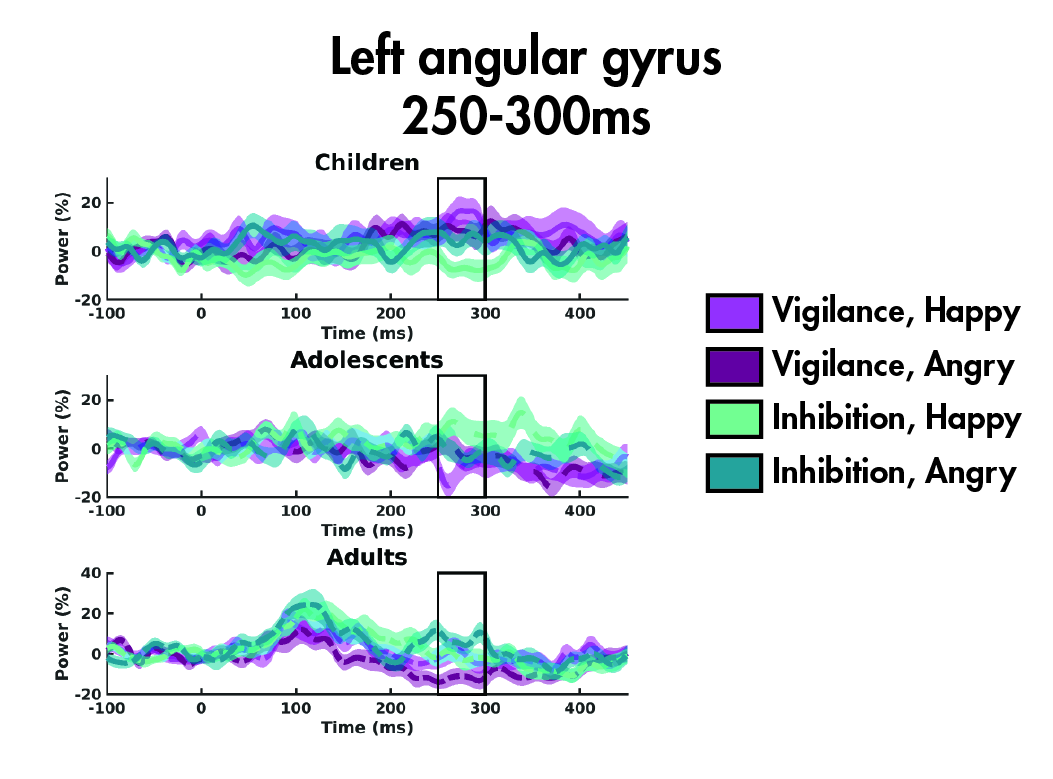


**Supplemental Figure 3**: Age group-by-condition-by-emotion interaction. The means and standard errors of power timeseries in each age group (children, adolescents and adults) for each emotion (vigilance: purple, inhibition: green; angry: darker shades, happy: lighter shades) are shown for angular gyrus.
